# Therapeutic Activation of PPARα Inhibits Transformed Follicular Lymphoma Tumorigenesis via the FOXM1 Signaling Pathway

**DOI:** 10.7150/ijbs.116437

**Published:** 2025-08-22

**Authors:** Dongmei Qin, Hui Zhou, Jie Zhou, Chendi Xie, Shuman Jia, Xingxing Yu, Yan Hong, Li Zhang, Yueting Huang, Yong Zhou, Bing Xu, Jie Zha

**Affiliations:** 1 Department of Hematology, The First Affiliated Hospital of Xiamen University and Institute of Hematology, School of Medicine, Xiamen University, Xiamen, China.; 2 Key Laboratory of Xiamen for Diagnosis and Treatment of Hematological Malignancy, Xiamen, 361003, China.; 3 Xiamen Hematology Medical Quality Control Center, Xiamen, China.; 4 State Key Laboratory of Cellular Stress Biology, Innovation Center for Cell Biology, School of Life Sciences, Xiamen University, Xiamen, China.

**Keywords:** Transformed follicular lymphoma, chiglitazar, PPARα, FOXM1

## Abstract

Transformed follicular lymphoma (t-FL) is a subtype of follicular lymphoma (FL) characterized by aggressive behavior and poor treatment outcomes. Dysregulated glucose uptake and cell cycle disruption have been implicated in t-FL progression. Here, we found that PPARα is frequently low-expressed in transformed follicular lymphoma, and therapeutic activation of PPARα significantly represses the progression of t-FL in cell line-derived xenograft (CDX) and primary t-FL patient-derived xenograft (PDX) models *in vivo*. Mechanistically, activation of PPARα inhibits t-FL progression mainly through three different signaling pathways as follows: PPARα inhibits glycolysis in t-FL cells by blocking the HIF1α signaling pathway; activation of PPARα induces mitochondria-dependent apoptosis in t-FL cells by disrupting mitochondrial homeostasis; and PPARα transcriptionally inhibits FOXM1 expression, causing the downregulation of its downstream target genes and inducing cell cycle arrest in t-FL cells. Concurrently, knockdown of FOXM1 enhances the sensitivity of t-FL cells to chiglitazar, and overexpression of FOXM1 partially rescued the inhibitory effect of chiglitazar on t-FL cells, highlighting the involvement of the PPARα-FOXM1 axis in the antitumor effects of chiglitazar. These promising preclinical results support further clinical evaluation of chiglitazar as a potential therapeutic option for t-FL patients, providing a novel and effective treatment approach for this aggressive subtype of FL.

## Introduction

Transformed follicular lymphoma (t-FL) is an aggressive histologic transformation of FL, predominantly into diffuse large B-cell lymphoma (DLBCL) 1. This transformation occurs in approximately 2% to 3% of FL patients each year 2, 3. Patients previously treated with chemoimmunotherapy for follicular lymphoma (FL) who subsequently experience transformation typically face unfavorable outcomes, with a median survival of less than 2 years 4, 5. Salvage chemotherapy followed by autologous stem cell transplantation (ASCT) is the current preferred treatment approach 6; however, it is not suitable for all patients, particularly elderly individuals or those with comorbidities that restrict their tolerance for intensive chemotherapy, making them ineligible for ASCT 7. While novel DLBCL treatments, such as bispecific antibodies and chimeric antigen receptor (CAR)-T cell therapies, are being investigated in t-FL patients 8, their limited selectivity and high-cost pose challenges to their widespread clinical use. Therefore, there is an urgent need to identify alternative therapeutic strategies that are effective, well tolerated, and more accessible for t-FL patients.

Tumors have a complex metabolic ecology that supports tumor progression through ATP production, maintenance of redox balance, and biosynthesis, in which the uptake and utilization of glucose and the homeostasis of the mitochondria are critical for tumor survival 9. Recent research has highlighted the importance of aberrant glucose metabolism and increased glucose uptake during the transformation of FL 10. Increased glucose metabolism is a hallmark of tumorigenesis and tumor cell growth in a complex tumor microenvironment, which could promote cancer cell survival and resistance to treatment by activating glycolysis-associated molecules and signaling pathways 11. Furthermore, metabolic reprogramming and cell cycle progression involve intricate molecular interactions in tumorigenesis 12. Nuclear translocation cascades of core glycolytic enzymes (PKM2/PFKFB3) generate self-reinforcing regulatory loops that mechanistically integrate metabolic adaptation with cell cycle checkpoints and oncogenic drivers (K-ras/c-Myc) 12, 13. Cell cycle dysregulation caused by mutations/deletions of CDKN2A and TP53 or MYC translocation/amplification in t-FL is one of the key factors in the histologic progression of FL to aggressive lymphoma 13, 14. Peroxisome proliferator-activated receptors (PPARs), including PPARα, δ, and γ, are important regulators of glucose homeostasis, integrating environmental signals and metabolic pathways, and they also regulate the cell cycle to influence tumor progression 15-17. Notably, aberrant expression of PPARα and PPARγ has been observed in malignant B cells, including non-Hodgkin's B cell lymphomas 18. However, the specific involvement of the PPAR pathway in glucose uptake, mitochondrial homeostasis, the cell cycle and cell survival in t-FL remains largely unexplored. Thus, combining pharmacological interference with tumor glycolysis, mitochondrial metabolism and the cell cycle is expected to be a therapeutic strategy for disrupting various metabolic compartments within tumors, and targeting PPARs may represent a promising therapeutic approach for addressing dysregulated glucose metabolism and the cell cycle in t-FL.

Chiglitazar, an orally available small molecule pan-PPAR agonist (i.e., simultaneous activation of PPARα, δ, and γ), has shown efficacy in the treatment of type 2 diabetes and nonalcoholic steatohepatitis 19. This study aimed to evaluate the preclinical efficacy and biological activity of chiglitazar in t-FL utilizing *in vitro* and *in vivo* models, including cell lines, primary t-FL cells, and animal xenografts. Specifically, chiglitazar inhibited t-FL cell proliferation both *in vivo* and *in vitro*. The tumor-suppressing activity of chiglitazar is likely attributable to the promotion of endogenous apoptosis in tumor cells by affecting glycolysis and mitochondrial homeostasis and the arrest of the cell cycle progression of tumors through the PPAR-FOXM1 signaling pathway. Notably, low doses of chiglitazar significantly induced a decrease in the mitochondrial membrane potential, an increase in reactive oxygen species (ROS), and mitochondrial dysfunction. Thus, chiglitazar may serve as an adjuvant drug for combination or sequential therapy of t-FL cells, providing a new and accessible therapeutic option for patients with t-FL, particularly those who are ineligible for ASCT or require alternative treatments owing to limited options. The present results may pave the way for the clinical evaluation and future implementation of chiglitazar in t-FL management.

## Materials and methods

### Chemical reagents

Chiglitazar, sourced from Chipscreen Biosciences Co., Ltd. (Shenzhen, Guangdong, China), was first dissolved in dimethyl sulfoxide (DMSO) and then prepared as a 100 mM stock solution. This solution was then stored at -80°C and further diluted to the indicated concentrations.

### Cell culture

RL, SC-1, Karpas 422 cell lines were obtained from the Institute of Hematology, Xiamen University School of Medicine (Xiamen, Fujian, China) and cultured in Roswell Park Memorial Institute 1640 medium (RPMI 1640, HyClone, Thermo Fisher Scientific, USA) supplemented with 10% FBS (PAN Seratech, Aidan Bach, Germany), 1% penicillin-streptomycin (1% P-S, NCM Biotech, #C100C5, China). The 293T cells were cultured in DMEM medium supplemented with 10% FBS and 1% P-S. These cell lines were cultured under standard conditions at a 37◦C incubator in a humidified atmosphere with 5% CO_2_.

### Primary follicular lymphoma samples

All procedures of the study were by the ethical standards of the Institutional Research Council and the Helsinki Declaration and were reviewed by The Ethical Review Committee of the First Affiliated Hospital of Xiamen University, with the informed consent of all the patients involved. In this study, lymphoma tissue was collected from 29 patients diagnosed with FL or t-FL in the Department of Hematology of the First Affiliated Hospital of Xiamen University. The clinical information of these patients is presented in** Supplementary Table S1**. Tumor tissues were minced and ground, then filtered through a 70-mesh cell strainer (Biosharp, #BS-70-XBS, China) and centrifuged to obtain the primary cells, which were cultured in RPMI-1640 medium containing 20% FBS and 1% P-S. Cell proliferation and apoptosis were detected after treatment with the indicated concentrations of chiglitazar according to the respective experimental requirements.

### Lentiviral transfection of transformed follicular lymphoma cells

*FOXM1*-specific short hairpin RNAs (shRNAs), FOXM1 expression plasmids, and control plasmids used in parallel experiments were procured from Miaoling Biological Engineering Co., Ltd. (Shanghai, China, #P71376, #P71377, #P60179, # P29436, # P30780). The above plasmids were co-transfected with lentiviral packaging plasmids (pMDL-Gag/Pol, pVSV-G, and pRev) in HEK-293T cells for 48 h. Stable transfected cell lines were selected using puromycin (1μg/mL, 72 h) and confirmed by Western blotting.

### Cell proliferation, cell death, and cell cycle analysis

The anti-proliferative activity of chiglitazar was determined by using the Cell Counting Kit-8 (CCK-8, MCE, Shanghai, China) and the absorbance at 450 nm was detected by an iMark™ Microplate Absorbance Reader (Bio-Rad, USA), and the doses corresponding to the 50% inhibitory concentration (IC_50_) were estimated as previously described 20. Apoptosis and cell cycle distribution were evaluated on cells treated with DMSO or different doses of chiglitazar, as previously reported 20.

### Colony formation assay

1 × 10^4^ /well cells at logarithmical growth phase were seeded in 24-well plates and treated with indicated concentrations of chiglitazar for 48 h. After washing, 500 cells /well were then cultured with complete methylcellulose medium in 12-well plates for 14 days at 37 °C, 5% CO_2_ with 95% humidity. Colonies consisting of at least 50 cells were counted and analyzed for colony-forming ability.

### JC-1 assay

2 × 10^5^/ml cells were seeded in 12-well plates and treated with the indicated concentrations of chiglitazar for 24 h. Mitochondrial membrane potential was detected by the Mitochondrial membrane potential assay kit with JC-1 (Beyotime, #C2006, China) and used according to the manufacturer's recommendations.

### Quantitative Real Time (QRT-PCR)

Total RNA was isolated from cells using TRIzol RNA Isolation Reagent (TransGen Biotech, # ET111-01, China) and reverse transcribed with ABScript III RT Master Mix for qPCR with gDNA Remover Kit (ABclonal, #RK20428, China) according to the manufacturer's instructions. QRT-PCR was performed using a 2X Universal SYBR Green FastqPCR Mix (ABclonal, #RK21203, China) and a CFX96 Touch Real-Time PCR Detection System (Bio-Rad, USA). Primers used for quantitative RT-PCR are listed in Supplemental Table S2. The relative expression levels of each gene were calculated as 2^-ΔΔCt^ after normalization to GAPDH/ACTIN.

### Western blotting

Protein extraction, separation, and immunoblotting were performed as previously described 20. The following antibodies were used: anti-PPARα (Abcam, Cambridge, MA, USA, #ab227074); anti-PPARγ (ABclonal, Wuhan, China, #A11183); anti-PPARD (ABclonal, Wuhan, China, #A5656); anti-FOXM1 (Abcam, Cambridge, MA, USA, #ab207298); anti-active+pro Caspase-3 (ABclonal, Wuhan, China, #A19654); anti-PARP (Cell Signaling Technology, #9532); anti-Cleaved-PARP (Cell Signaling Technology, #5625); anti-Bcl-XL ( ABclonal, Wuhan, China, #A0209); anti-MCL-1 (Cell Signaling Technology, #4572 ); anti-CyclinB1 ( Huabio, Hangzhou, China, #ET1608-27); anti-C-MYC (Cell Signaling Technology, #18583); anti-CyclinD1 (Cell Signaling Technology, #55506); anti-CyclinE1 (Cell Signaling Technology, #4129); anti-GAPDH (Huabio, Hangzhou, China, #ET1601-4); anti-COX IV (Cell Signaling Technology, #4850); anti-Cytochrome c (Cell Signaling Technology, #4280); anti-HSP60 (Cell Signaling Technology, #12165); anti-PHB1 (Cell Signaling Technology, #2426); anti-Pyruvate Dehydrogenase (PHD) (Cell Signaling Technology, #3205); anti-SDHA (Cell Signaling Technology, #11998); anti-SOD1 (Cell Signaling Technology, #4266); anti- HIF1α (Abcam, #ab51608); Glycolysis Antibody Kit (include:anti-HIF1A; anti-HK2; anti-PKM2; anti-PFKP; anti-LDHA; anti-GLUT1; anti-PGK1) (Huabio, Hangzhou, China, #HAK21008); anti-CDK1 (proteintech, wuhan, China, #19532-1-AP); anti-CDK2 (proteintech, wuhan, China, #10122-1-AP).

### Chromatin immunoprecipitation (ChIP) assay

The ChIP assay was performed as previously described 21. Briefly, 1 × 10^7^ RL or SC-1 cells were cultured in a 100-mm cell culture dish and treated with 20 μM chiglitazar for 48 h. RL or SC-1 cells were collected, and the Simple ChIP Enzymatic Chromatin IP kit (Cell Signaling Technology, #9003) was used to perform the ChIP assay. In this procedure, DNA is fragmented by sonication, and anti-PPARα antibody (Abcam, Cambridge, MA, USA, #ab227074) or rabbit IgG is added with ChIP-Grade Protein G Magnetic Beads and incubated overnight at 4 °C to immunoprecipitate the DNA-containing complexes. DNA was isolated and detected by quantitative real-time PCR using the *FOXM1* promoter-specific primers listed below:

Forward, 5′-gtcacgtgaccttaac-3′; Reverse, 5′-caccggagctttcag-3′.

### Luciferase reporter assay

1 × 10^5^ cells /well 293T were cultured in 12-well plates and co-transfected with 100 ng PGL3-FOXM1-LUC and PCMV-3 × FLAG-PPARα plasmids (0 ng, 200 ng, 400 ng, and 800 ng, respectively) using Hieff Trans® Liposomal Transfection Reagent to test the transcriptional activity of the *FOXM1* gene promoter. A loading control of 20 ng of Renilla luciferase plasmid was employed. After 36 h of transfection, the cells were harvested, and luciferase activity was measured using the Dual-Luciferase reporter assay system (Promega Corp., Madison, WI, USA).

### Immunohistochemical (IHC) staining

For IHC staining, a Mouse/Rabbit Probe HRP Labeling Kit with DAB Brown (BIOTnA, China) was used according to the manufacturer's instructions, as described previously 20. Primary antibodies included anti-Ki67 (#A20018, ABclonal, Wuhan, China; 1:200), and anti-PCNA (#A9909, ABclonal, Wuhan, China; 1:200).

### RNA sequencing analysis

Briefly, 1×10^6^ SC-1 cells were seeded in 10-cm dishes, treated with chiglitazar for 24 h, harvested by centrifugation, washed with PBS, snap-frozen in liquid nitrogen, and the RNA sequencing were performed by Seqhealth Technology Co., Ltd. Transcriptomic analysis identified differentially expressed genes (DEGs; |fold change| > 1.5, p < 0.05), visualized through volcano plots and hierarchical clustering heatmaps. Subsequent functional annotation included KEGG pathway enrichment analysis and Gene Set Enrichment Analysis (GSEA) to elucidate biological processes.

### Glucose and lactate measurements

Briefly, RL, SC-1, and Karpas 422 cells were seeded at 2×10^5^ cells/well in 12-well plates and treated with gradient concentrations of chiglitazar for 24 h. The supernatants were collected for metabolic analysis: cellular glucose uptake was quantified using an o-toluidine method-based Glucose Assay Kit (Beyotime Biotechnology, China; #S0201M), while lactate production was determined using a specialized Lactic Acid Assay Kit (Nanjing Jiancheng Bioengineering Institute, China; #A019-2-2).

### Seahorse metabolic analysis

Briefly, RL and SC-1 cells (2×10⁵ per well) were seeded in 24-well plates and treated with chiglitazar for 24 h. Cells were counted and reseeded (2×10⁴ per well) onto poly-L-lysine-coated XF96-well culture plates. Extracellular acidification rate (ECAR) and oxygen consumption rate (OCR) were quantified using the Seahorse XF Glycolysis Stress Test Kit (Agilent Technologies, Santa Clara, CA, #103020-100) and Seahorse XF Cell Mito Stress Test Kit (Agilent Technologies, Santa Clara, CA, #103015-100), respectively, following manufacturer protocols.

### Data sources

The GSE86613 microarray dataset (Johnsen HE et al.) was retrieved from the Gene Expression Omnibus (GEO) database (https://www.ncbi.nlm.nih.gov/geo/). Following robust multiarray analysis (RMA) with background adjustment and quantile normalization, differentially expressed genes (DEGs) were identified (adjusted p < 0.05, |log2FC|≥ 1.5). Functional annotation was performed through the Kyoto Encyclopedia of Genes and Genomes (KEGG) pathway analysis and Gene Set Enrichment Analysis (GSEA).

### Animal studies

The CB17/Icr-Prkdc^scid^/IcrlcoCrl (CB-17 SCID) mice (5 weeks old) used in this study were purchased from Xiamen University Animal Care and maintained in a pathogen-free environment. All animal experiments were approved by the Laboratory Animal Ethics and Management Committee of Xiamen University.

To establish cell line-derived xenograft (CDX) mouse models, 1 Gy-irradiated 5-week-old female CB-17 SCID mice were subcutaneously injected with 1 × 10^7^ RL or SC-1 cells. Once the tumor's longest diameter reached 3 mm, a 10-day treatment regimen of 15 mg/kg chiglitazar was initiated. Tumor volume, tumor weight, and body weight were continuously monitored and analyzed throughout the treatment period.

To establish patient-derived xenograft (PDX) mouse models, 1 Gy-irradiated 5-week-old female CB-17 SCID mice were subcutaneously injected with 1 × 10^7^ primary cells derived from lymph node tissue of patients with t-FL. When the longest diameter of the tumor reached 1.5 cm, the first generation of PDX cells was harvested to reconstruct the PDX mouse models. In analogy, we established the PDX mouse models with fifth-generation PDX cells, and the subsequent process was the same as that in the CDX mouse models.

### Statistical analysis

All values are means ± SD of at least three replicates unless otherwise indicated. All analyses were carried out using GraphPad Prism 7.0 software. Variables between the two groups were compared using Student's t-test. Comparisons among multiple groups were performed with the one-way or two-way ANOVA. *P* < 0.05 was considered statistically significant.

## Results

### Transformed follicular lymphoma is characterized by aberrant cell cycle regulation and abnormal glucose metabolism

**Figure 1:**
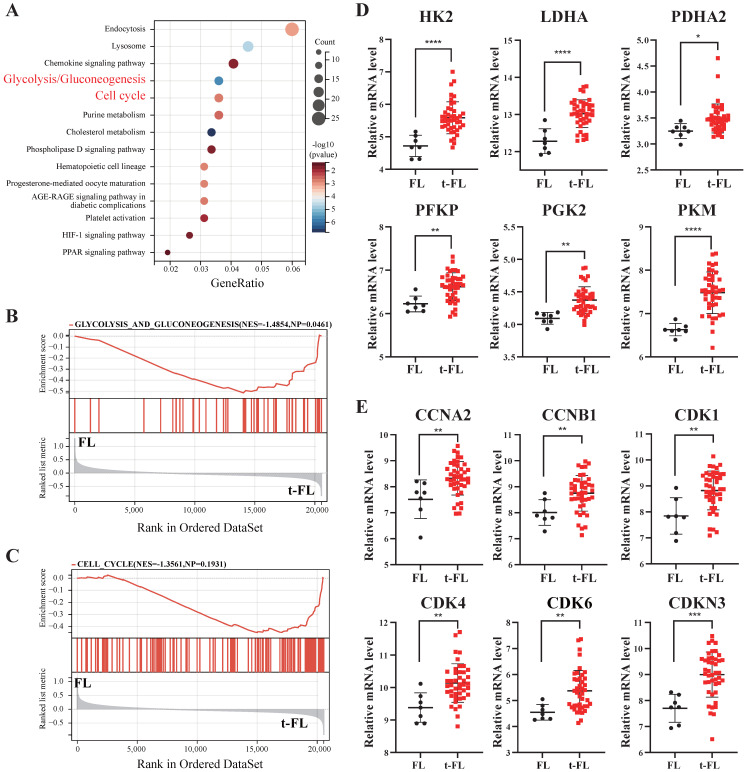
** Transcriptomic interrogation of cell cycle regulation and glucose metabolism in transformed follicular lymphoma.** (**A-C**) KEGG **(A)** and GSEA** (B-C)** enrichment analysis of GSE86613 data enriched to glycolysis/glycogenesis (NES=1.4854) and cell cycle (NES=1.3561) pathways. (**D**) Transcriptomic profiling of GSE86613 revealed marked upregulation of glycolysis/gluconeogenesis pathway -related genes (*HK2*, *LDHA*, *PDHA2*,* PFKP*, *PKG2*, and *PKM*) expression in t-FL specimens. (**E**) Transcriptomic interrogation of GSE86613 demonstrated significant upregulation of core cell cycle related genes (*CCNA2*, *CCNB1*, *CDK1*, *CDK4*, *CDK6*, and *CDKN3*) expression in t-FL specimens. (*P < 0.05, **P < 0.01, ***P < 0.001, ****P < 0.0001).

Previous studies have demonstrated that metabolic abnormalities, increased glucose uptake, and cell cycle disorders have crucial effects on the progression of lymphomas 22. Therefore, to elucidate the significance of metabolic and cell cycle profiling in t-FL progression, we analyzed transcriptome profiling data from FL and t-FL samples (GSE86613). KEGG analysis and GSEA revealed that the glycolysis/gluconeogenesis and cell cycle pathways were significantly enriched in the progression of t-FL (**Figure 1A-C**). Moreover, glycolysis-related genes (*HK2*, *LDHA*, *PDHA2*,* PFKP*, *PKG2*, and *PKM*) and cell cycle-related genes (*CCNA2*, *CCNB1*, *CDK1*, *CDK4*, *CDK6*, and *CDKN3*) were highly expressed in t-FL samples (**Figure 1D-E**). Taken together, these results suggested that the upregulation of glycolysis and disruption of the cell cycle are involved in the progression of t-FL.

### Chiglitazar increases the expression of PPARα and has cytotoxic effects on transformed follicular lymphoma cells *in vitro* and *in vivo*

**Figure 2:**
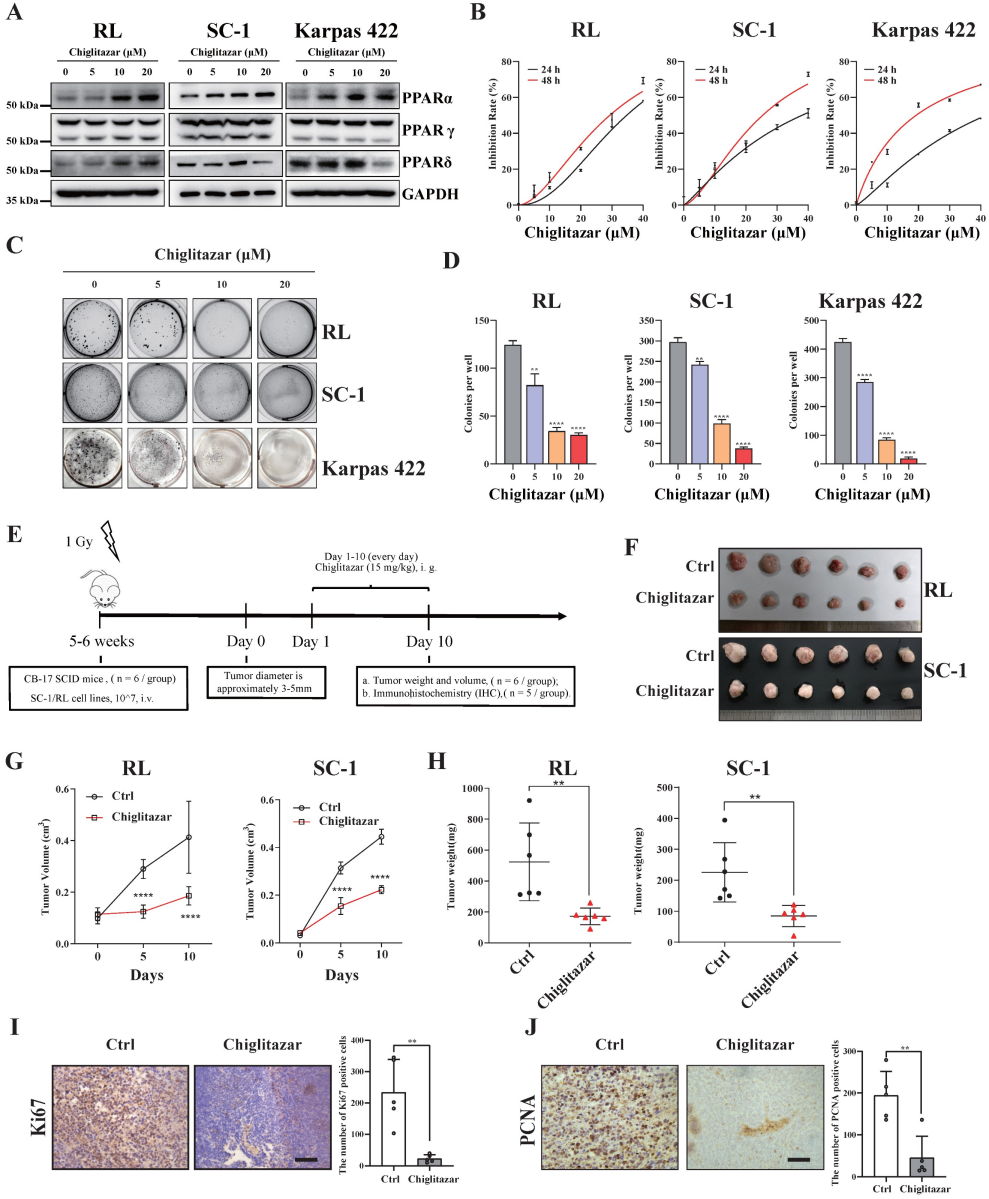
**Chiglitazar increases the expression of PPARα and exhibits cytotoxic effects on transformed follicular lymphoma cell lines both *in vitro* and *in vivo*.** (**A**) Western blotting was performed to analyze the protein expression levels of PPARα, PPARγ, and PPARδ in RL, SC-1 and Karpas 422 cells after being treated with DMSO or chiglitazar (5 μM, 10 μM, 20 μM) for 24 h. (**B**) RL, SC-1 and Karpas 422 cells were treated with DMSO or chiglitazar (10 μM, 20 μM, 30 μM, 40 μM) for 24 and 48 hours respectively, and then the cell viability was tested by CCK-8 assay. (**C-D**) Chiglitazar synergistically inhibited cell colony formation of RL, SC-1 and Karpas 422 cells, and colony formation was measured by colony formation assay after treatment with DMSO or chiglitazar (5 μM, 10 μM, 20 μM) for 48 hours. (**E**) Schematic outline of the CDX models. (**F-H**) CB17/SCID mice were treated with chiglitazar or vehicle control. Volume (**F-G**) and weight (**H**) of tumors were measured and calculated. Values are means ± SEM for 6 mice/each group. (**I-J**) The expression of Ki67 (**I**) and PCNA (**J**) in t-FL CDX xenograft tissues was examined by IHC analysis (scale bar: 50μm), and the number of cells positively stained with Ki67 and PCNA was calculated for each image from three independent fields. (*P < 0.05, **P < 0.01, ***P < 0.001, ****P < 0.0001).

Chiglitazar, a hypoglycemic agent, regulates glucose‒lipid metabolism, and our previous study demonstrated that chiglitazar has a lethal effect on AML cells 20. In the present study, we also found that PPARα, a target of chiglitazar, was expressed at lower levels in t-FL samples than in normal CD19^+^ B cells from healthy donors (**Supplementary Figure S1**). Moreover, in the RL, SC-1, and Karpas 422 t-FL cell lines, chiglitazar significantly increased PPARα protein levels in a dose-dependent manner but did not affect PPARβ or PPARγ protein levels (**Figure 2A**). These results suggested that chiglitazar has a potential antitumor effect on t-FL.

The antiproliferative efficacy of the pan-PPAR agonist, chiglitazar, was next evaluated in the RL, SC-1 and Karpas 422 t-FL cell lines. As assessed by CCK-8 assays performed after 24 h and 48 h of chiglitazar exposure, chiglitazar reduced the viability of RL, SC-1, and Karpas 422 cells in a time- and dose-dependent manner (**Figure 2B**; median IC50 values are shown in **Supplementary Table S3**). A colony formation assay was also conducted to evaluate the inhibitory effect of chiglitazar on the proliferation of t-FL cells. As depicted in **Figure 2C and Figure 2D,** chiglitazar substantially diminished the number of colonies formed by RL, SC-1, and Karpas 422 cells in a dose-dependent manner.

The *in vivo* ability of chiglitazar to reduce tumor growth was assessed in CB17/SCID mice injected with t-FL cells. The CDX model was generated via the subcutaneous injection of RL and SC-1 cell lines into CB17/SCID mice (**Figure 2E**). Compared with the control, chiglitazar, which was administered at a dose of 15 mg/kg daily, significantly affected tumor growth, as manifested by decreases in tumor weight and tumor size (**Figure 2F, Figure 2G, Figure 2H**). Compared with the control treatment, chiglitazar treatment was well tolerated and did not result in body loss in the RL and SC-1 CDX models (**Supplementary Figure S2**). To further validate cell proliferation within the subcutaneous graft tumors, immunohistochemical analysis was performed to assess the expression of the Ki67 and PCNA proliferation-related markers. As shown in **Figure 2I** and **Figure 2J**, the chiglitazar-treated CDX models presented notably fewer Ki67- and PCNA-positive cells than the vehicle-treated control mice. Taken together, these results suggested that chiglitazar is effective against t-FL both *in vitro* and* in vivo* and that it is well tolerated.

### Chiglitazar significantly modulates multiple signaling pathways in transformed follicular lymphoma cells

**Figure 3:**
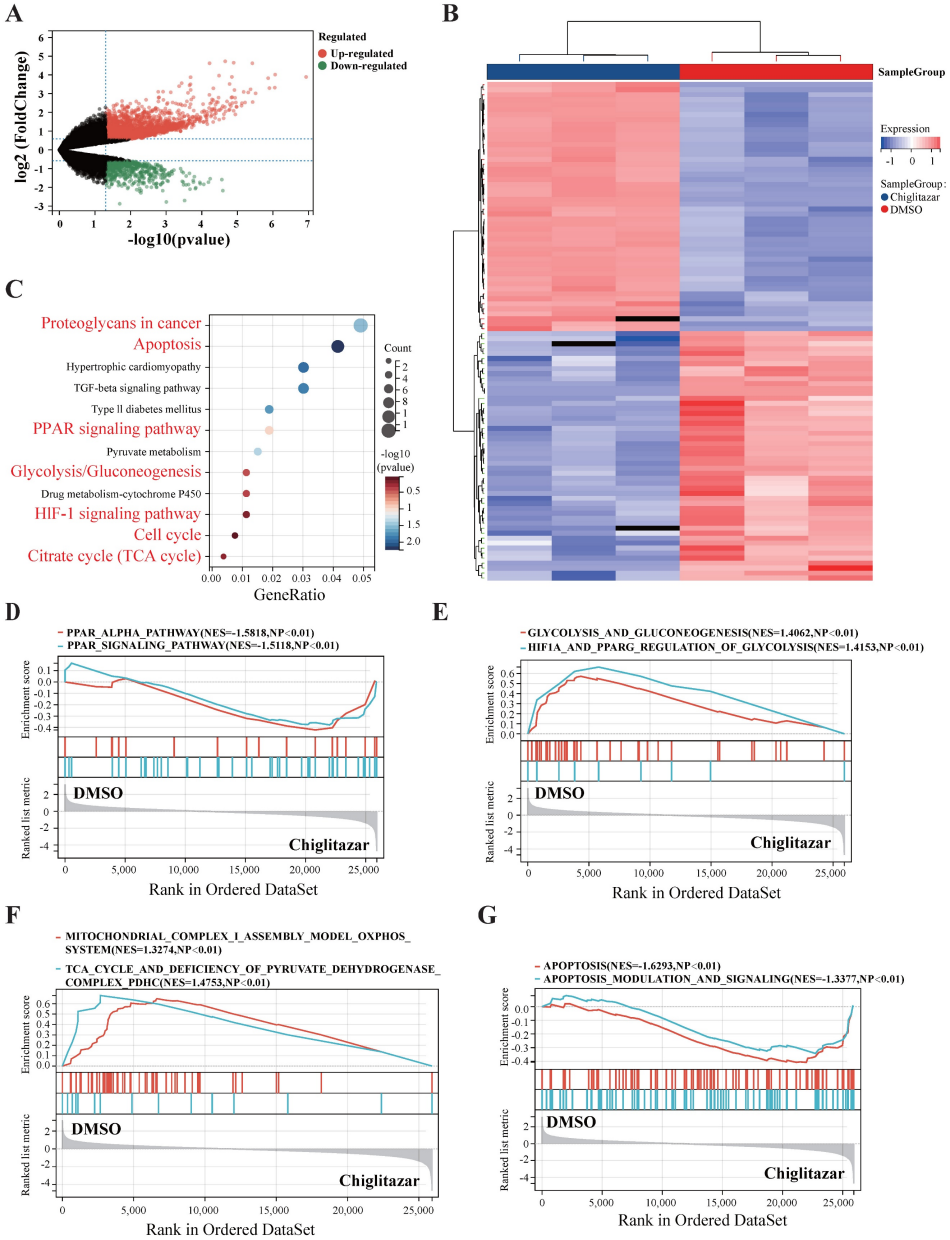
** RNA-seq was used to analyze the transcriptional profiles of transformed follicular lymphoma cells treated with chiglitazar.** (**A-B**) Volcano plot (**A**) highlights key targets (red: upregulation; green: downregulation), and hierarchical clustering heatmap (**B**) delineates distinct expression patterns of top 50 DEGs of RNA sequencing data from SC-1 cells after 24 h of chiglitazar treatment. (**C**) KEGG enrichment analysis demonstrated that multiple signaling pathways were enriched in chiglitazar-treated t-FL, including apoptosis and glycolysis/gluconeogenesis. (**D-G**) GESA analysis revealed that multiple signaling pathways were enriched in chiglitazar-treated t-FL cells, including PPARα signaling (**D**), glycolytic pathways (**E**), mitochondrial functional regulation (**F**), and apoptotic pathways (**G**). (*P < 0.05, **P < 0.01, ***P < 0.001, ****P < 0.0001).

To explore the molecular mechanisms by which chiglitazar suppresses t-FL progression, SC-1 cell lines were treated with chiglitazar, and RNA-seq was performed for transcriptomic profiling. The volcano plot and heatmap data analysis revealed 3,268 differentially expressed genes (2,412 upregulated; 856 downregulated) in the transcriptomic profile (**Figure 3A, Figure 3B**). KEGG analysis and GSEA revealed that chiglitazar modulated PPARα-related (**Figure 3C, Figure 3D**), glucose metabolism-related (**Figure 3C, Figure 3E**), cell cycle-related (**Figure 3C**), mitochondrial function-related (**Figure 3C, Figure 3F**), and apoptosis-related (**Figure 3C, Figure 3G**) pathways. These data suggested that chiglitazar affects the antitumor efficacy of t-FL cells by modulating multiple signaling pathways.

### Chiglitazar significantly inhibits the glycolysis pathway in transformed follicular lymphoma cells

**Figure 4:**
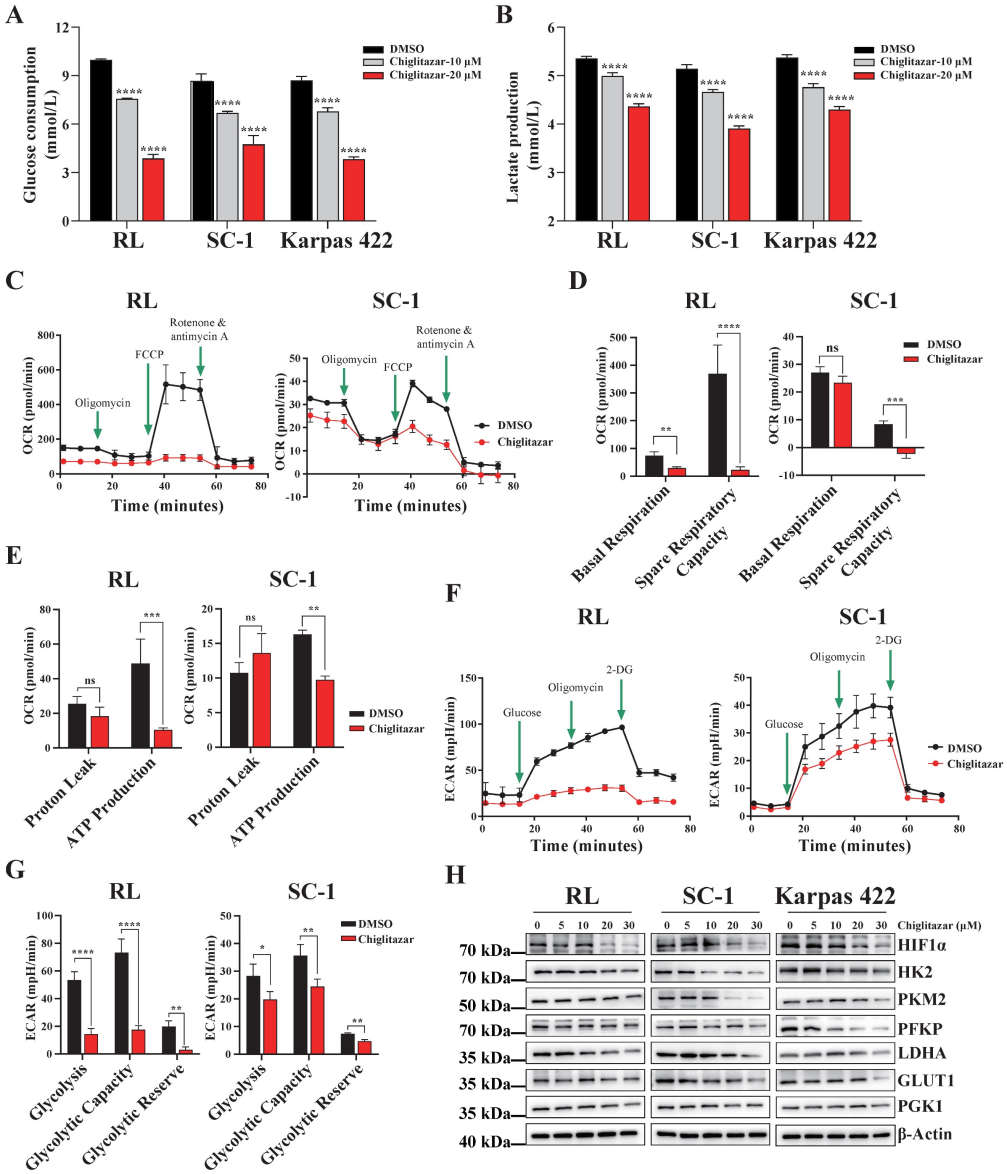
** Chiglitazar remarkably suppresses the glycolytic pathway in transformed follicular lymphoma cells.** (**A-B**) Glucose consumption (**A**) and lactate production (**B**) were inhibited in RL, SC-1 and Karpas 422 cells after 24 h of chiglitzar (10 μM, 20 μM) treatment, and the glucose consumption and lactate production were measured using the Glucose Assay Kit and Lactic Acid Assay Kit, respectively. (**C-E**) OCR levels in RL and SC-1 cells after 24 h of 20 μM chiglitazar treatment (**C**) in which mitochondrial Spare Respiratory Capacity (**D**) and ATP Production (**E**) were impaired. (**F-G**) ECAR levels in RL and SC-1 cells following 24 h of 20 μM chiglitazar (**F**) exposure in which glycolytic activity (**G**) was damaged. the ECAR and OCR were quantified using the Seahorse XF Glycolysis Stress Test Kit and Seahorse XF Cell Mito Stress Test Kit, respectively. (**H**) The levels of glycolysis-associated proteins (HIF1α, HK2, PKM2, PFKP, LDHA, GLUT1 and PGK1) were analyzed by Western blotting after 24 h of exposure to chiglitazar (5 μM, 10 μM, 20 μM, 30 μM). (*P < 0.05, **P < 0.01, ***P < 0.001, ****P < 0.0001).

Cancer cells prefer to metabolize glucose via glycolysis even in the presence of sufficient oxygen 23, and PPARα is a key transcription factor involved in glucose metabolism 20, 24. On the basis of the transcriptomic profiling data, chiglitazar-mediated regulation of the glycolysis pathway in t-FL cells was investigated. Chiglitazar significantly decreased glucose uptake and lactate production in RL, SC-1 and Karpas 422 cells (**Figure 4A, Figure 4B**). To further characterize the glycolytic pathway mediated by chiglitazar in t-FL cells, the oxygen consumption rate (OCR) and extracellular acidification rate (ECAR) of the RL and SC-1 cell lines treated with chiglitazar were measured. As shown in **Figure 4C, Figure 4D, and Figure 4E,** chiglitazar significantly decreased the OCR in RL and SC-1 cells, and it also reduced the ECAR (**Figure 4F, Figure 4G**). To understand the mechanism by which chiglitazar inhibits glycolysis in t-FL cells, the expression of several key glycolytic proteins and HIF1α, an effector in the HIF1 signaling pathway, was examined. As shown in **Figure 4H,** chiglitazar decreased the protein levels of HIF1α, HK2, PKM2, PFKP, LDHA, GLUT1, and PGK1. Taken together, these results suggested that chiglitazar inhibits glycolysis in t-FL cells by suppressing the expression of glycolysis-related proteins, including HIF1α, HK2, PKM2, PFKP, LDHA, GLUT1, and PGK1.

### Chiglitazar induces intrinsic-mediated apoptosis in transformed follicular lymphoma cells by disrupting mitochondrial homeostasis

**Figure 5:**
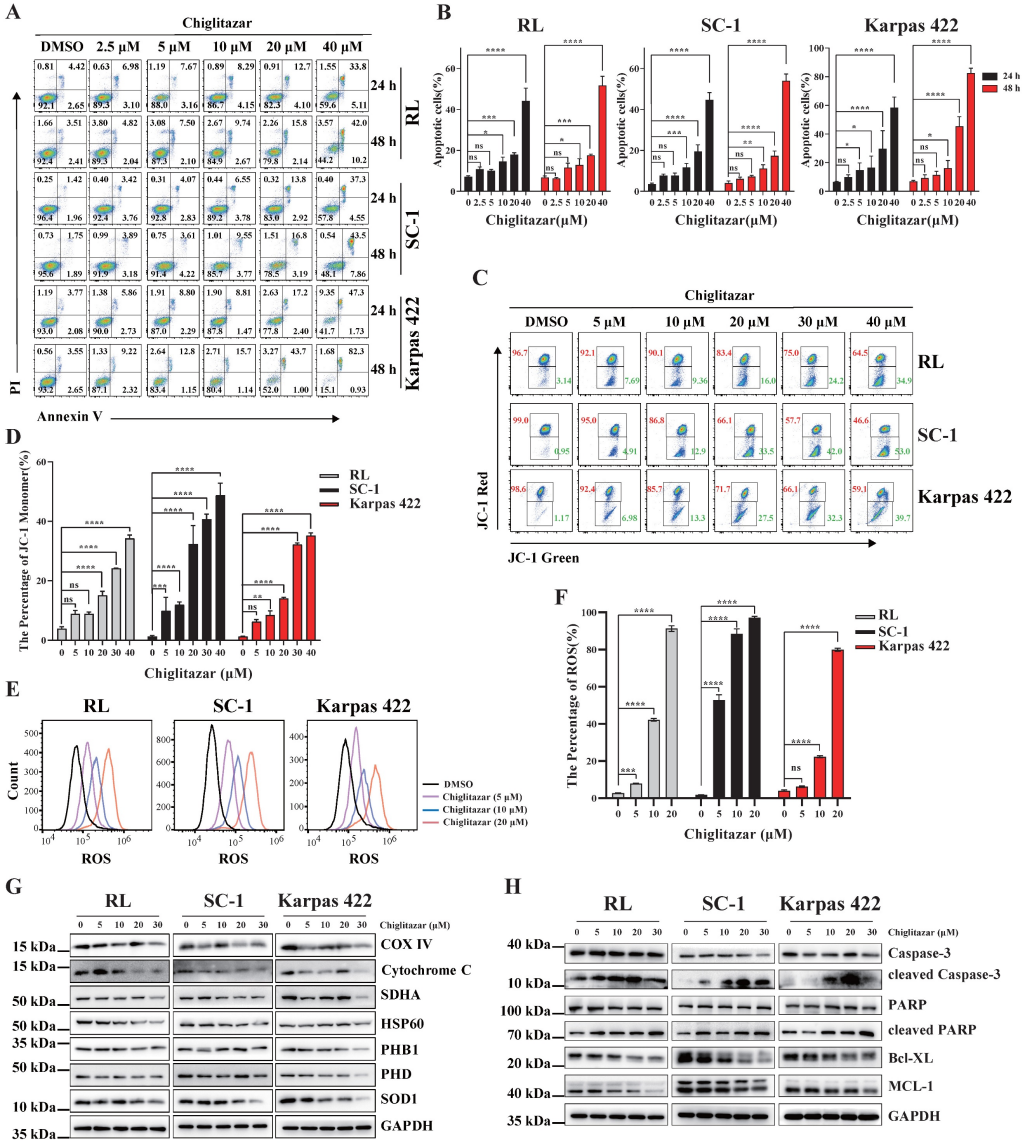
** Chiglitazar significantly induced intrinsic-mediated apoptosis level in transformed follicular lymphoma cells.** (**A-B**) Representative images **(A)** and statistical results **(B)** of apoptosis in RL, SC-1 and Karpas 422 cells treated with DMSO or chiglitazar (2.5 μM, 5 μM, 10 μM, 20 μM, 40 μM) for 24 h and 48 h, and cell apoptosis was measured by Annexin V/PI staining. (**C-D**) Representative images (**C**) and statistical results (**D**) of Mitochondrial Membrane Potential (MMP) in RL, SC-1 and Karpas 422 cells treated with DMSO or chiglitazar (5 μM, 10 μM, 20 μM, 30 μM, 40 μM) for 24 h, and the MMP was measured by JC-1 staining. (**E-F**) Chiglitazar significantly enhanced Reactive Oxygen Species (ROS) levels in RL, SC-1 and Karpas 422 cells, and the ROS levels was measured by DCFH-DA (2,7-Dichlorodi-hydrofluorescein diacetate) staining in RL, SC-1 and Karpas 422 cells treated with DMSO or chiglitazar (5 μM, 10 μM, 20 μM) for 24 h. **(G-H)** The levels of mitochondrial-related proteins (COX Ⅳ, cytochrome C, SDHA, HSP60, PHB1, and PDH) **(G)**, and mitochondrial apoptosis-associated proteins (cleaved Caspase-3, cleaved PARP, BCL-XL, and MCL-1) (**H**) were analyzed by Western blotting under different concentrations of chiglitazar (5 μM, 10 μM, 20 μM, 30 μM) treatment for 24 h. (*P < 0.05, **P < 0.01, ***P < 0.001, ****P < 0.0001).

Transcriptome profiling data suggested that apoptosis-related pathways were enriched in chiglitazar-treated t-FL cells (**Figure 3C, Figure 3G**). Annexin V/PI staining and flow cytometry were performed to investigate whether chiglitazar induces apoptosis in t-FL cells. After exposure to chiglitazar for 24 h and 48 h, the percentage of apoptotic RL, SC-1, and Karpas 422 cells significantly increased in a dose-dependent manner. Specifically, the percentages of apoptotic RL, SC-1, and Karpas 422 cells exceeded 50% upon treatment with 40μM chiglitazar (**Figure 5A and Figure 5B**). Given that the loss of the mitochondrial membrane potential (MMP) is pivotal for triggering the intrinsic mitochondria-dependent apoptotic cascade 25, the impact of chiglitazar on the MMP was investigated. Chiglitazar led to a reduction in the MMP in RL, SC-1, and Karpas 422 cells, as evidenced by the increase in the percentage of JC-1 monomers (**Figure 5C and Figure 5D**). A reduction in the mitochondrial membrane potential results in a mitochondrial redox imbalance, which impacts mitochondrial balance and functionality 26. The effect of chiglitazar on the level of ROS was subsequently measured in t-FL cells. The ROS levels were significantly elevated after chiglitazar treatment, suggesting that mitochondrial homeostasis may have been disturbed (**Figure 5E and Figure 5F**). In addition, the expression of mitochondria-associated proteins, such as COX IV, cytochrome C, SDHA, HSP60, PHB1, and pyruvate, was downregulated after chiglitazar treatment (**Figure 5G**). Furthermore, the expression of apoptosis-related proteins associated with the mitochondrial pathway was evaluated. Chiglitazar treatment upregulated the protein expression of cleaved caspase-3 and cleaved PARP but downregulated the protein expression of BCL-XL and MCL-1 in RL, SC-1, and Karpas 422 cells (**Figure 5H**). Collectively, these results indicated that chiglitazar decreases the mitochondrial membrane potential and increases ROS levels in t-FL cells, leading to disruption of mitochondrial homeostasis, which mediates mitochondria-dependent apoptosis.

### Chiglitazar induces cell cycle arrest by blocking the FOXM1 signaling pathway

**Figure 6:**
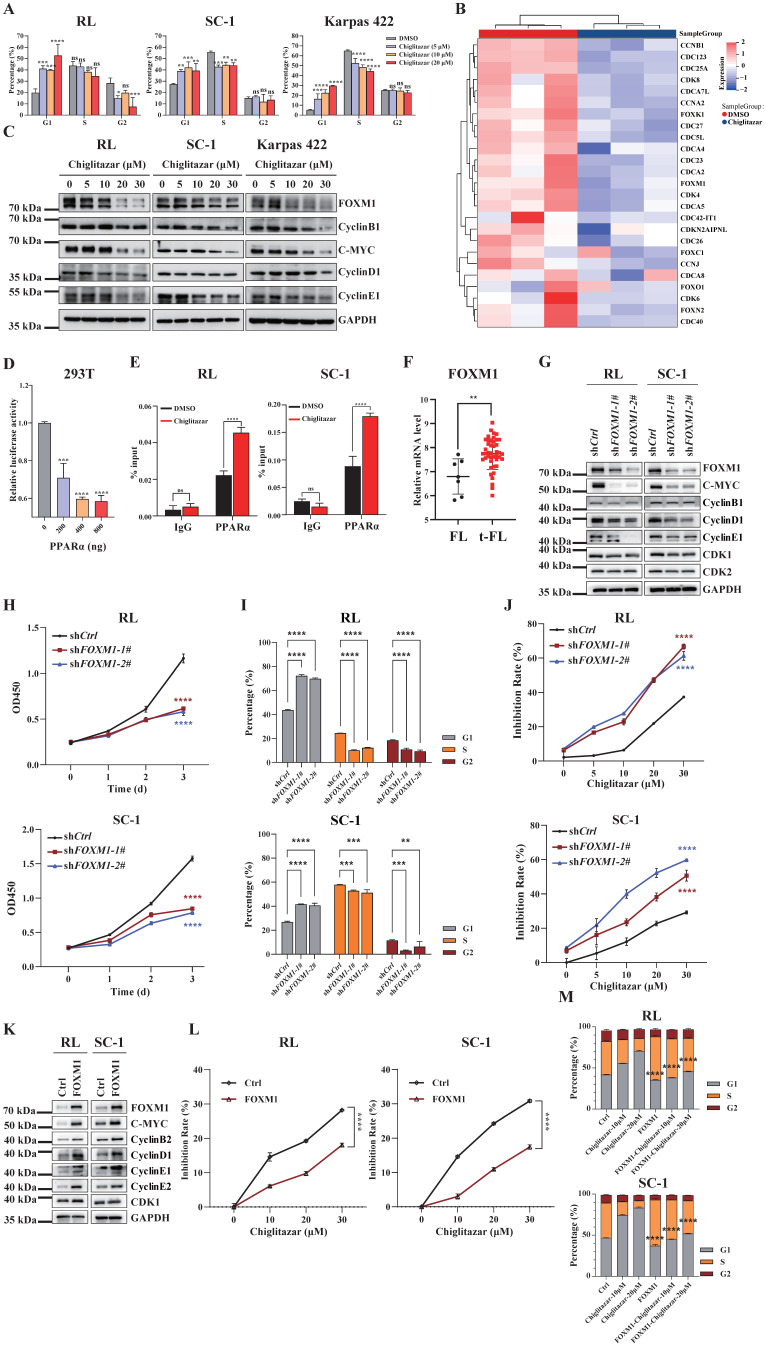
**Chiglitazar induces cell cycle arrest by blocking the FOXM1 signal pathway in transformed follicular lymphoma cells.** (**A**) The cell cycle of RL, SC-1, and Karpas 422 was arrested in the G1 phase after being treated with chiglitazar (5 μM, 10 μM, 20 μM) for 24 h. (**B**) Heatmap analysis showed that the expression of cell cycle-related genes was down-regulated in SC-1 cells after being treated with 20 μM chiglitazar for 24 h, including *FOXM1* and its downstream target genes (such as *CCNB1*,* CCNA2* and *CDK4*). **(C)** Western blotting was used to analyze the expression of cell cycle-associated proteins (FOXM1, C-MYC, cyclin B1, cyclin D1, and cyclin E1) in RL, SC-1, and Karpas 422 cells treated with chiglitazar (5 μM, 10 μM, 20 μM, 30 μM) for 24 h.** (D)** Overexpression of PPARα downregulated the *FOXM1* gene promoter reporter activity in 293T cells.** (E)** The ChIP assay performed to detect the enrichment of PPARα on the *FOXM1* gene promoter region in RL and SC-1 cells after treatment with 20 μM chiglitazar for 24 h. **(F)** Transcriptome analysis of GSE86613 showed that *FOXM1* expression was upregulated in t-FL specimens.** (G)** The *FOXM1*-knockdown RL and SC-1 cell lines was constructed by lentiviral system, and the expression of FOXM1, C-MYC, CyclinB1, CyclinD1, CyclinE1, CDK1 and CDK2 was measured by western blotting assay. **(H-I)** Knockdown of *FOXM1* inhibited cell viability **(H)** and induced cell cycle arrest **(I)** in RL and SC-1 cells, cell viability and cell cycle detected by CCK-8 and PI staining assays, respectively.** (J)** Knockdown of *FOXM1* significantly enhanced the sensitivity of RL and SC-1 cells to chiglitazar. **(K)** The *FOXM1*-overexpressed RL and SC-1 cell lines was constructed by lentiviral system, and the expression of FOXM1, C-MYC, CyclinB2, CyclinD1, CyclinE1, CyclinE2, and CDK1 was measured by western blotting assay. **(L)** Overexpression of *FOXM1* impairs the inhibitory effect of chiglitazar on cell viability of RL and SC-1 cells, the cell viability measured by CCK8 assay in *FOXM1*-overexpressed RL and SC-1 cells treated with chiglitazar (10 μM, 20 μM, 30 μM) for 24 h. **(M)** Overexpression of *FOXM1* significantly attenuates the cell cycle arrest effect of chiglitazar on RL and SC-1 cells, and the cell cycle measured by PI staining assay in wild type- or *FOXM1* overexpressed- RL and SC-1 cells treated with chiglitazar (10 μM, 20μM) for 24 h. (*P < 0.05, **P < 0.01, ***P < 0.001, ****P < 0.0001).

Aberrant cell cycle regulation is a key factor in tumorigenesis and in the histological progression of FL to aggressive lymphoma 13. As the transcriptomic profiling data revealed that cell cycle-related pathways were enriched in t-FL cells treated with chiglitazar (**Figure 3C**), cell cycle analysis was performed using chiglitazar-treated t-FL cells. Chiglitazar treatment of RL, SC-1, and Karpas 422 cells resulted in an increased proportion of cells in the G1 phase and decreased proportions of cells in the G2 and S phases (**Figure 6A** and **Supplementary Figure S3**). FOXM1 is an oncogenic transcription factor that is widely dysregulated in various tumors and is involved in cell proliferation, cell survival, and cell cycle regulation 27, 28. Heatmap analysis revealed that the expression of *FOXM1* and its downstream cycle-related genes was significantly downregulated in chiglitazar-treated t-FL cells (**Figure 6B**). To investigate whether chiglitazar induces cell cycle arrest by suppressing the FOXM1 signaling pathway in t-FL cells., RL, SC-1, and Karpas 422 cells were treated with chiglitazar, and the expression of FOXM1 and its downstream cell cycle-related genes was measured by western blot and quantitative real-time PCR analyses. As shown in **Figure 6C**, chiglitazar significantly decreased the protein expression levels of FOXM1 and its downstream cell cycle-related proteins, namely, C-MYC, cyclin B1, cyclin D1, and cyclin E1, in a dose-dependent manner. The mRNA expression levels of FOXM1 and its downstream cell cycle-related genes (C-MYC, cyclin D1, cyclin E1, CDK1, CDC25C, cyclin B1, and cyclin A2) were also decreased (**Supplementary Figure S4**). Together, these results suggested that chiglitazar induces t-FL cell cycle arrest by inhibiting the FOXM1 signaling pathway.

Because chiglitazar effectively activated PPARα expression in the RL, SC-1, and Karpas 422 cell lines (**Figure 2A),** luciferase reporter and ChIP assays were performed to evaluate whether PPARα directly regulates the transcriptional level of *FOXM1*. PPARα repressed the transcriptional activity of the *FOXM1* promoter, and chiglitazar effectively enhanced the recruitment of PPARα to the *FOXM1* gene promoter region in RL and SC-1 cells (**Figure 6D, Figure 6E** and **Supplementary Figure S5)**. Taken together, these results suggested that chiglitazar-activated PPARα transcriptionally represses FOXM1 expression.

To determine whether FOXM1 serves as the pivotal mediator of the antitumor effects of chiglitazar in t-FL cells, transcriptome analysis was performed, which revealed that *FOXM1* expression was upregulated in t-FL samples compared with FL samples (**Figure 6F**). Next, *FOXM1*-knockdown RL and SC-1 cell lines were established using shRNA, which demonstrated a significant reduction in the expression of downstream genes, such as C-MYC, cyclin B1, cyclin D1, cyclin E1, CDK1, and CDK2 (**Figure 6G and Supplementary Figure S6**). Moreover, *FOXM1* knockdown significantly inhibited proliferation, arrested the cell cycle, and increased the sensitivity of RL and SC-1 cells to chiglitazar (**Figure 6H, Figure 6I, Figure 6J and Supplementary Figure S7A**). Correspondingly, overexpression of *FOXM1* significantly increased the expression of downstream genes (C-MYC, CyclinB2, CyclinD1, CyclinE1, CyclinE2, and CDK1) in RL and SC-1 cell lines (**Figure 6K**), and it also significantly attenuated the inhibitory effect of chiglitazar on RL and SC-1 cells viability (**Figure 6L**) as well as cell cycle blocking effect (**Figure 6M and Supplementary Figure S7B**). Taken together, these results underscored the pivotal role of FOXM1 in t-FL cell proliferation and its potential to increase the therapeutic impact of chiglitazar on t-FL cells.

### Chiglitazar exhibits cytotoxic effects on a patient-derived t-FL xenograft mouse model

**Figure 7:**
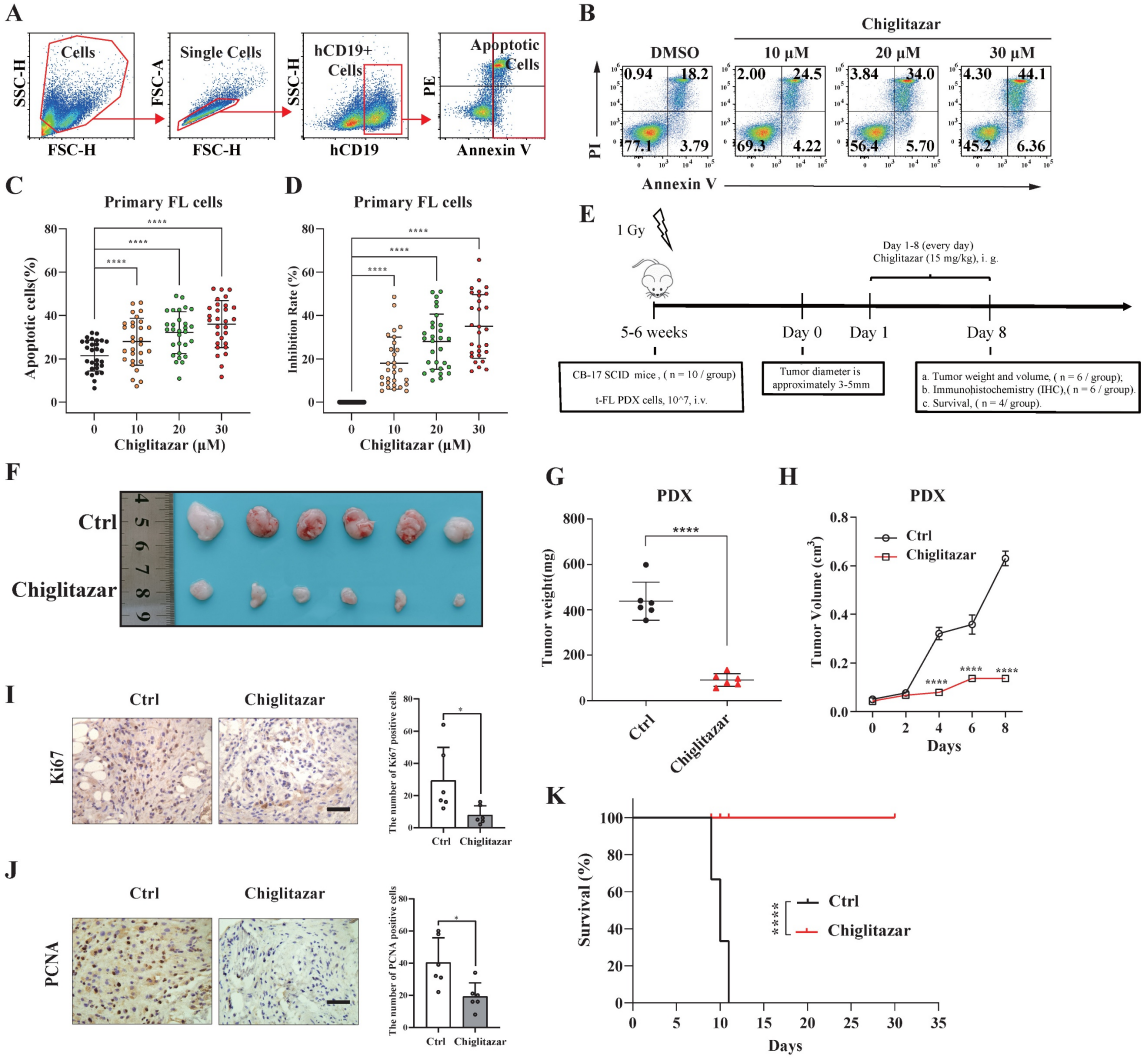
** Chiglitazar inhibits tumorigenesis in a t-FL patient-derived xenograft mouse model.** (**A-C**) Flow cytometric assessment of apoptosis in primary t-FL patient-derived cells (n=29) following chiglitazar treatment 24-hour: gating strategy **(A)**, representative images **(B)**, and statistical results **(C)**. **(D)** Cell viability of primary cells from t-FL patients (n=29) after chiglitazar (0 μM, 10 μM, 20 μM, 30 μM) treatment for 24 hours by CCK-8 assay. **(E)** Schematic outline of the t-FL PDX models. (**F-H**) The PDX model mice treated with chiglitazar or vector control. Weight (**F-G**) and volume (**H**) of tumors were measured and calculated. Values are means ± SEM for 6 mice/each group. (**I-J**) The expression of Ki67 (**I**) and PCNA (**J**) in t-FL PDX xenograft tissues was examined by IHC analysis (scale bar: 50μm). The number of cells positively stained with Ki67 and PCNA was calculated from three independent fields for each image. (**K**) Kaplan-Meier survival curve of patient-derived xenograft mice treated with chiglitazar or vehicle. (*P < 0.05, **P < 0.01, ***P < 0.001, ****P < 0.0001).

The impact of chiglitazar on primary t-FL cells was next investigated. After exposure of primary samples (n=29) to increasing doses of chiglitazar for 24 h, cell apoptosis was evaluated via flow cytometry assessment using APC-Annexin V, PI and FITC-CD19 staining (**Figure 7A**). Notably, chiglitazar induced cell apoptosis in primary t-FL samples in a dose-dependent manner (**Figure 7B-7C**). Furthermore, chiglitazar decreased the viability of primary t-FL samples (**Figure 7D**). These findings validated the cytocidal effects of chiglitazar on primary t-FL cells.

A PDX mouse model was utilized to investigate the *in vivo* antitumor potential of chiglitazar (**Figure 7E**). PDX cells were identified by flow cytometry using human CD10, CD19, and CD20 antibodies. As shown in **Supplementary Figure S8**, these PDX cells presented specific antigenic features on their surface, mirroring the characteristics of t-FL. Upon treatment with chiglitazar at a dosage of 15 mg/kg/day, tumor growth in response to t-FL was significantly suppressed, as evidenced by significant decreases in tumor size and weight (**Figure 7F-7H**). Immunohistochemistry analysis revealed a significant reduction in Ki-67 and PCNA expression in the chiglitazar-treated mice compared with the control mice (**Figure 7I**-**7J**). Moreover, chiglitazar significantly prolonged the survival rate of the PDX model mice (**Figure 7K**). Together, these results highlighted the efficacy of chiglitazar in alleviating t-FL burden.

## Discussion

The transformation of follicular lymphoma into a more aggressive subtype, known as transformed FL (t-FL), is associated with a poorer prognosis and limited treatment options 3. Clinically, t-FL presents with aggressive features, such as prominent B symptoms (fever, night sweats, and unintentional weight loss), elevated LDH levels, frequent extranodal tissue involvement, and a 5-year survival of only 20-30% 29. Currently, the treatment strategy for patients with t-FL who have received prior chemotherapy is similar to that for patients with *de novo* diffuse large B cell lymphoma (DLBCL), which typically involves intensive chemotherapy and stem cell transplantation (SCT), depending on patient fitness 30. CAR-T cell therapy has emerged as a promising option for t-FL patients 3. However, limited efficacy and frequent relapses are observed with intensive therapy, and SCT or cellular therapy is only suitable for fit patients. These limitations necessitate further exploration of alternative and more effective therapeutic strategies for t-FL. Chiglitazar, which is approved for the treatment of type 2 diabetes mellitus, has been demonstrated to hinder the growth of cancer cells by interfering with multiple metabolic pathways 20, 31. The present study highlights the ability of chiglitazar, a PPAR agonist, to exhibit preclinical efficacy against t-FL. Our findings indicate the broad anti-proliferative and cytotoxic activity of chiglitazar in both t-FL cell lines and primary t-FL patient samples. Additionally, the present data elucidate the ability of chiglitazar to curtail t-FL cell growth in xenograft mouse models. Interestingly, our previous studies showed no significant pharmacotoxicity of chiglitazar on healthy donor-derived hematopoietic stem cells, and these studies emphasize the safety of chiglitazar in the blood system 21,32. Given the complex pathogenesis of t-FL, future investigations should delve into potential combination therapies involving chiglitazar to increase treatment efficacy while minimizing adverse effects.

To improve the treatment of t-FL, it is crucial to gain a better understanding of the mechanisms underlying disease progression and transformation from the indolent phase to the aggressive phase. Several genetic and biological features have been identified that may explain the aggressiveness and chemoresistance of t-FL, including *TP53* mutations and/or deletions in 15-30% of transformed cases, *B2M* mutations and/or deletions in 20-25% of transformed cases, *CDKN2A/B* deletions in 20-30% of transformed cases, and *MYC* translocations 14. Recurrent alterations in genes involved in the control of cell cycle progression and the DNA damage response suggest that loss of gene stability and dysregulated proliferation are critical steps in the development of t-FL. Thus, targeting the cell cycle may serve as an effective strategy for the management of t-FL.

The wide-ranging effectiveness of chiglitazar against both t-FL cell lines and primary patient samples suggests its impact on a signaling pathway commonly dysregulated in aggressive B cell lymphomas. Among the key regulators of multiple t-FL processes is the FOXM1 transcription factor. The present study revealed a substantial decrease in FOXM1 transcriptional levels upon chiglitazar treatment, which was attributed primarily to the activation of PPARα. Notably, FOXM1 is highly expressed in nearly 80% of DLBCL patients, with prior research emphasizing its central role in promoting tumor progression 33, 34. Increased FOXM1 expression is closely linked with advanced tumor staging, a high proliferation rate, and an unfavorable prognosis, highlighting its potential as a novel prognostic marker for cancer patients 34. Consistent with its role in regulating the cell cycle, chiglitazar-induced FOXM1 inhibition triggered G1 phase arrest, reduced the proportion of cells in the G2/M phase, and downregulated cell cycle-related proteins, such as C-MYC, cyclin B1, cyclin D1, and cyclin E1. As FOXM1 is a critical mediator of tumor cell survival, its suppression likely explains the observed reduction in colony-forming ability following chiglitazar treatment. FOXM1 suppression not only inhibited t-FL cell proliferation but also alleviated chiglitazar-induced cell death. Conversely, overexpression of FOXM1 significantly promoted t-FL cell proliferation. These results reveal the pro-tumorigenic function of FOXM1 in t-FL progression and establish it as a key molecular target. Since FOXM1 plays a key role in cell proliferation and chemotherapy sensitivity, targeting FOXM1 represents a promising therapeutic strategy for t-FL.

Inherited heterogeneity exists in most tumors, with diverse activated oncogenes and loss of tumor suppressor genes; alterations in many oncogenes and tumor suppressor genes induce a common metabolic phenotype, and treatments based on this common feature may be a better anticancer strategy than therapies based on the complex and highly variable genetic profiles of tumors 9. Metabolic dissimilarity persists between tumor cells and normal cells, with tumor cells consuming significantly more glucose; in addition, tumor cells also retain functional mitochondria and oxidative phosphorylation ability, and targeted depletion of mitochondrial DNA reduces the tumorigenic potential of cancer cell lines *in vivo* and *in vitro* 35. Interestingly, metabolic reprogramming during disease progression in B cell lymphomas has been proposed as a mechanism 10, 36. There is growing evidence that metabolic reprogramming not only confers cell growth and survival advantages to tumor cells but also affects antitumor immunity 37. In t-FL, increased expression of the glycolytic machinery is associated with transformation, as evidenced by the increased glucose uptake detected by ^18^F-FDG PET/CT 38. However, the role and mechanism by which PPARα regulates glycolysis in t-FL are not clear. In this study, we report that glycolytic dysfunction induced by PPARα activation is another important mechanism by which chiglitazar exerts antitumor effects on t-FL. Specifically, glucose consumption, lactate production, OCR, and ECAR assays revealed that chiglitazar activates PPARα and significantly inhibits glycolysis, whereas western blot analysis revealed that chiglitazar suppresses glycolysis in t-FL cells by downregulating the expression of HIF1α and its downstream glycolysis-associated proteins (HK2, PKM2, PFKP, LDHA, GLUT1, and PGK1).

Given the abnormal glucose metabolism observed in t-FL cells, targeting tumor glycolysis and mitochondrial homeostasis may be a potential therapeutic strategy. PPARα (also known as NR1C1), a ligand-activated nuclear receptor, has been associated with cell survival in cancer because of its effects on glycolytic metabolic pathways and lipid metabolism in cancer cells 39, 40. Previous studies have demonstrated that PPARα deficiency promotes colon carcinogenesis in mice by increasing DNMT1-mediated p21 methylation and PRMT6-mediated p27 methylation 41. Moreover, emerging evidence suggests that PPARα inhibits tumor progression by regulating multiple signaling pathways, making it a potential therapeutic target for cancer treatment 42-44. While the important functions and mechanisms of PPARs in various metabolic processes have been well elucidated, their role in t-FL remains poorly understood. On the basis of the present findings, we propose that metabolic disruption and mitochondrial redox dysfunction induced by PPARα activation may be among the mechanisms underlying the antitumor effect of chiglitazar in t-FL. Specifically, in t-FL cells, chiglitazar primarily activates PPARα, decreases the mitochondrial membrane potential, increases the level of ROS, downregulates the expression of mitochondria-related proteins (such as COX IV, cytochrome C, SDHA, HSP60, PHB1, and pyruvate), and disrupts the redox balance of the mitochondria, causing mitochondrial dysfunction and inducing mitochondria-dependent apoptosis.

The regulation of redox homeostasis is fundamental to maintaining normal cellular function and ensuring cell survival, and cancer cells exhibit persistently high levels of ROS as a result of genetic, metabolic, and microenvironmental-related alterations, which are counteracted by the enhancement of antioxidant defense mechanisms in cancer cells 45. However, the dependence of cancer cells on their antioxidant system represents a specific vulnerability, against which tumor cell death can be induced by increasing oxidative stress above the toxicity threshold without affecting normal cells 46. As metabolic hubs, mitochondria and ROS are the nexus of multiple pathways that determine the cellular response to intracellular homeostatic disruptions, and excessive or inappropriately localized ROS can damage cells and affect apoptosis, stem cell differentiation, and autophagy 47. Our research revealed that in t-FL cells, low-dose chiglitazar markedly elevates ROS, reduces mitochondrial protein levels, and disturbs the redox equilibrium. On the basis of these findings, we believe that chiglitazar has promising clinical application in t-FL and can be used in combination or in a sequential manner with other chemotherapies.

## Conclusion

In summary, the present study unequivocally establishes chiglitazar as a potent PPAR agonist with remarkable antitumor efficacy in both cell-based assays and animal models of t-FL. Chiglitazar, which specifically activates PPARα, interferes with glycolysis and redox homeostasis and hinders the FOXM1 signaling pathway in t-FL cells *in vitro*, ultimately causing cell proliferation inhibition, cell death, and cell cycle arrest. Chiglitazar also has antitumor effects on tumor xenografts without altering the weight of the mice, which partly explains its tolerability in mice. Collectively, our preclinical investigation underscores the efficacy and safety of chiglitazar, thereby warranting future clinical assessments of chiglitazar, especially in combination with other chemotherapeutic agents, for the treatment of t-FL.

## Supplementary Material

Supplementary figures and tables.
