# Supplementary material for: Therapeutic Activation of PPARα Inhibits Transformed Follicular Lymphoma Tumorigenesis via the FOXM1 Signaling Pathway
Source: Int J Biol Sci. 2025 Aug 22;21(12):5411–27. doi: 10.7150/ijbs.116437 (PMC12435485; doi:10.7150/ijbs.116437)
Supplement: Supplementary file 1 — Supplementary figures and tables. [file ijbsv21p5411s1.pdf]

Supplemental Data

Supplemental Figures

Supplemental Figure S1

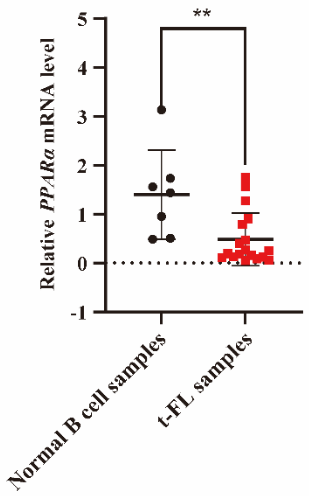

PPARα is low-expressed in t-FL samples compared to normal CD19$^+$ B cells from healthy donors.

Supplemental Figure S2

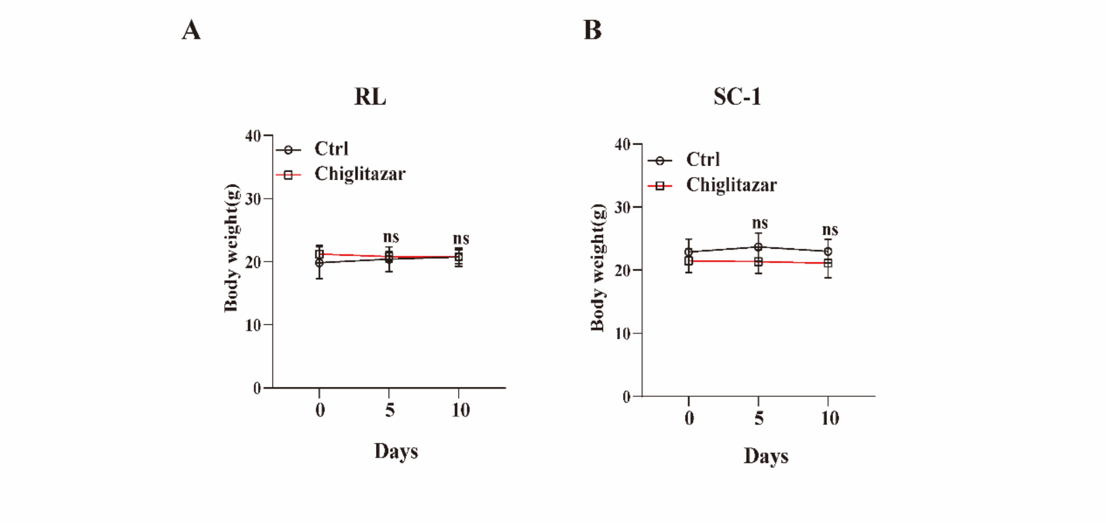

The body weights of the CDX mice of RL **(A)** and SC-1 **(B)** cells did not significantly change after administration of chiglitazar (15 mg/kg).

Supplemental Figure S3

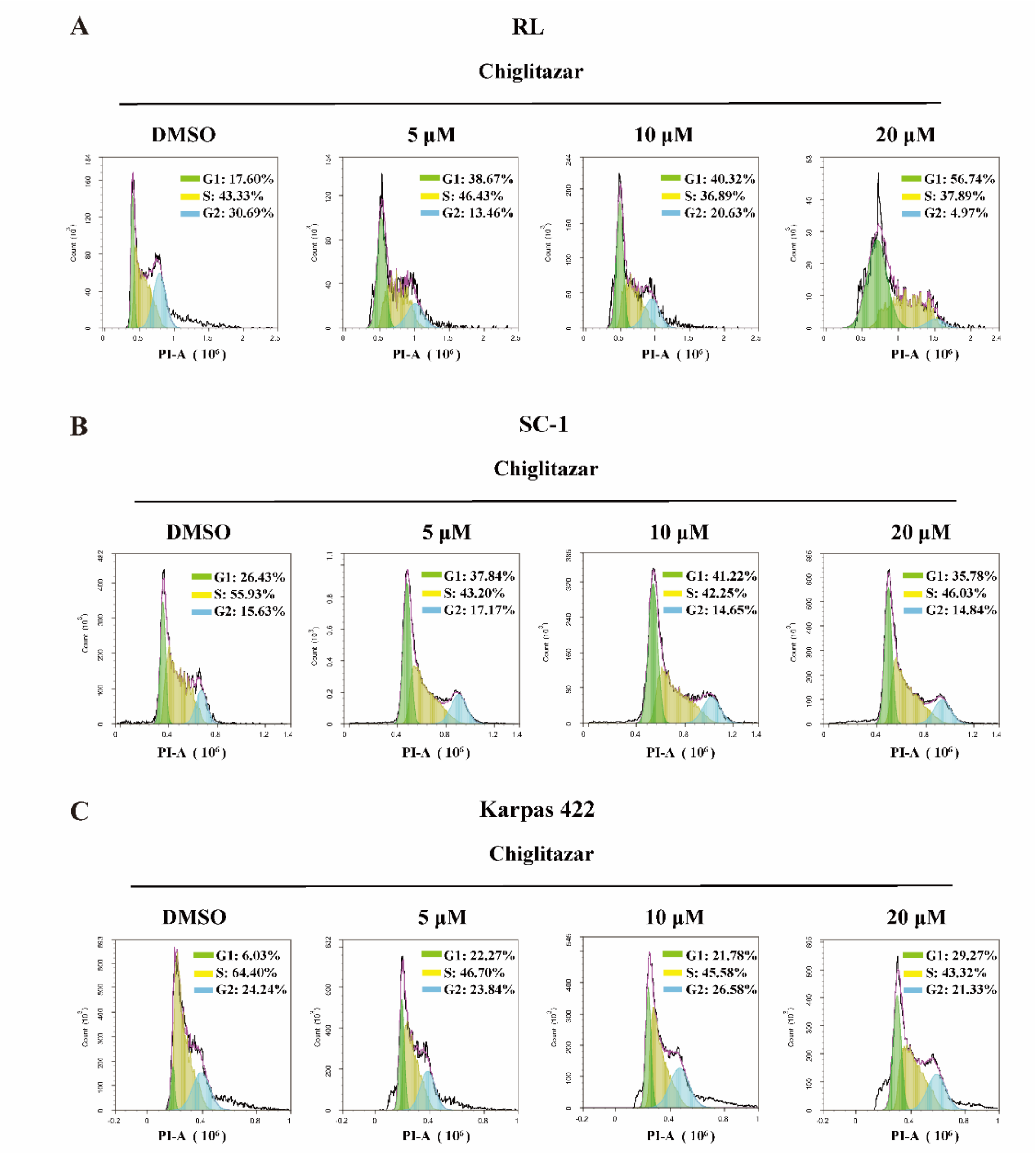

Representative images of the cell cycle of RL (A), SC-1 (B), and Karpas 422 cells (C) after treatment with DMSO or chiglitazar (5 μM, 10 μM, 20 μM) were analyzed by PI staining.

Supplemental Figure S4

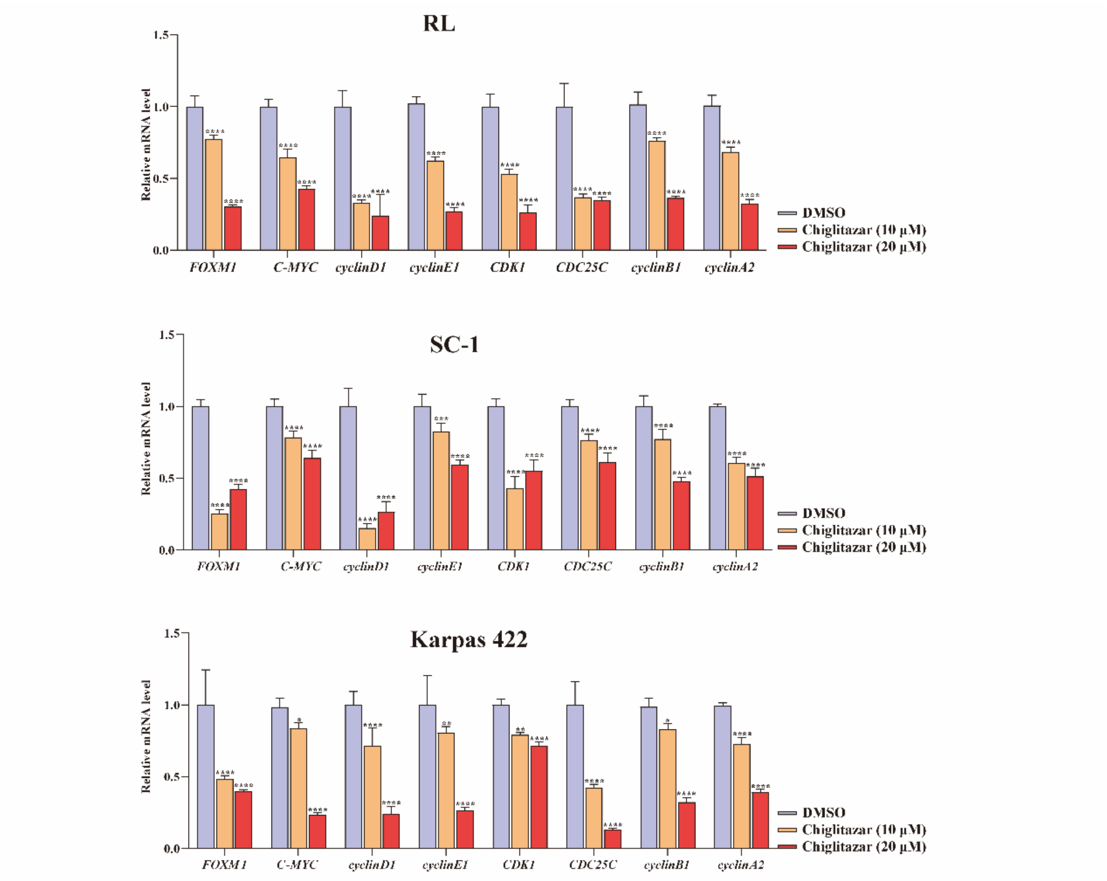

Real-time PCR analysis was used to analyze the effects of chiglitazar treatment (10 μM, 20 μM) for 24 h on the mRNA levels of RL, SC-1, and Karpas 422 cell cycle-related genes (*FOXM1*, *C-MYC*, *cyclin B1*, *cyclin D1*, and *cyclin E1*).

Supplemental Figure S5

A

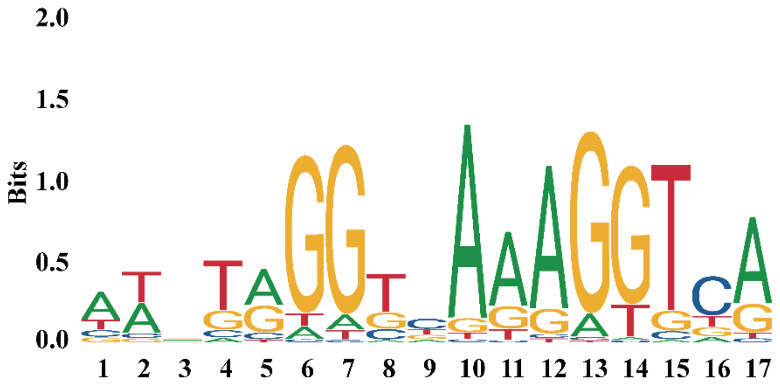

B

Matrix ID	Name	Score	Relative score
MA1147.1	MA1147.1.NR4A2::RXRA	9.10522	0.826577122
MA1146.1	MA1146.1.NR1H4::RXRA	10.3195505	0.825526832
MA1146.1	MA1146.1.NR1H4::RXRA	10.020849	0.820851085
MA1147.1	MA1147.1.NR4A2::RXRA	8.082063	0.812364993
MA0159.1	MA0159.1.RARA::RXRA	10.386636	0.809478649

The PPARα binding site in the promoter region of *FOXM1* was predicted using the Jasper website. Motif analysis of the accessible promoter region **(A)** and relative scores for different regions **(B)**.

Supplemental Figure S6

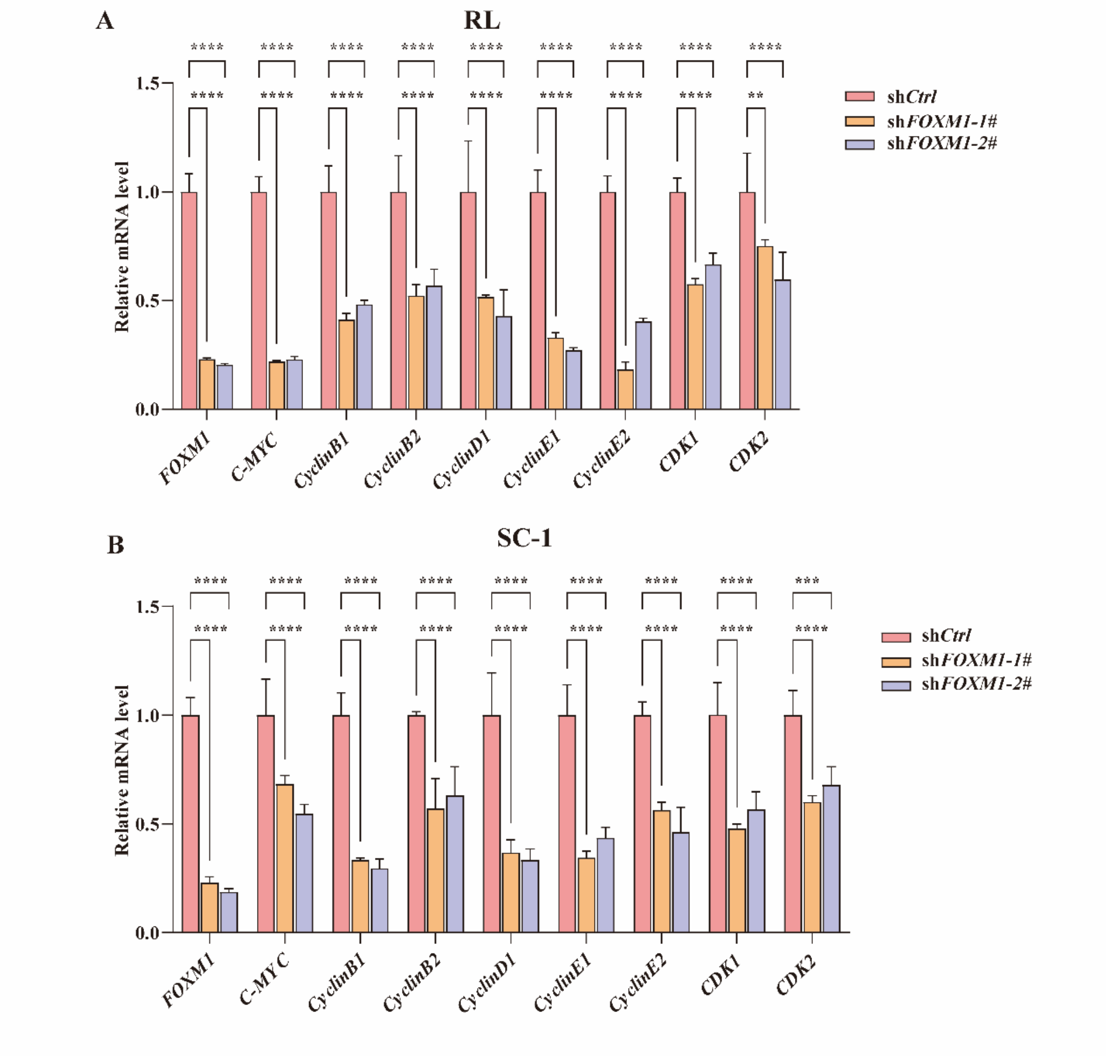

Expression of *FOXM1* and its target genes (*C-MYC*, *CyclinB1*, *CyclinD1*, *CyclinE1*, *CyclinE2*, *CDK1* and *CDK2*) was assessed by real-time PCR in *FOXM1*-knockdown RL **(A)** and SC-1 cells **(B)**.

Supplemental Figure S7

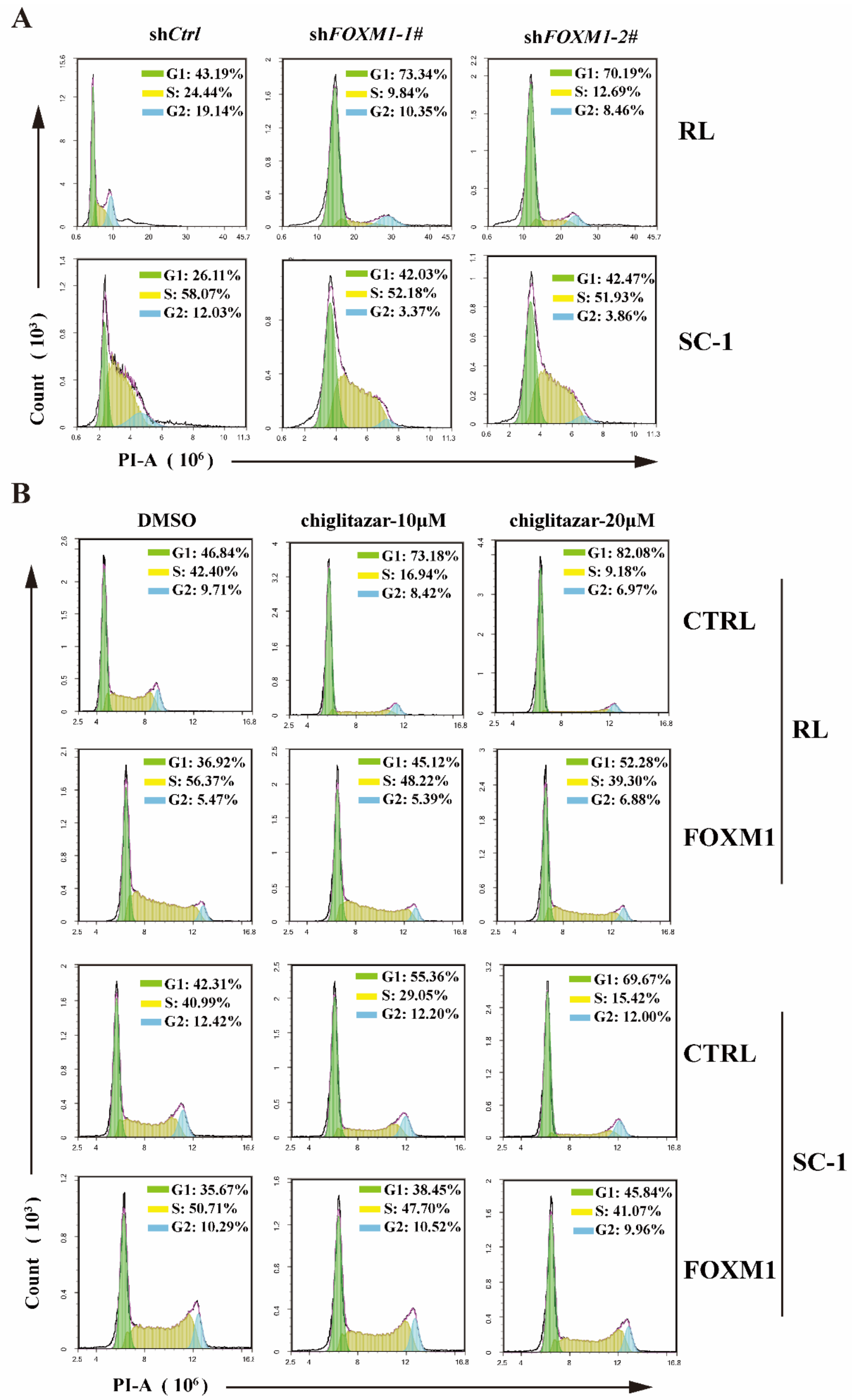

Representative images of the cell cycle in *FOXM1*-knockdown **(A)** and *FOXM1*-overexpressed **(B)** RL and SC-1 cells were analyzed using PI staining.

Supplemental Figure S8

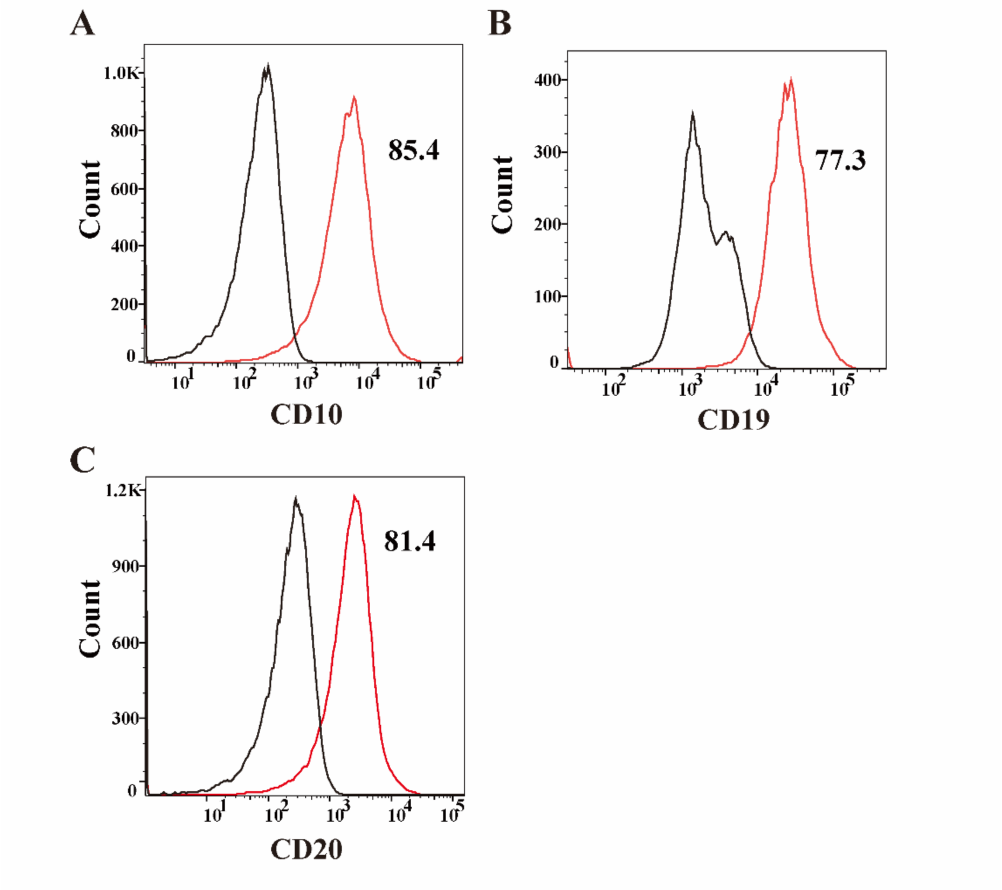

The t-FL PDX cells are identified by flow cytometry staining using human CD10, CD19 and CD20 antibodies.

Supplementary Tables

Supplementary Table 1. Clinical characteristics of FL and t-FL patients.

Clinical characteristics of FL and t-FL patients

Patient No	Clinical diagnosis	Stage	relapse	Age	Gender
1	Follicular lymphoma	unknown	no	64	F
2	Follicular lymphoma	III	no	58	M
3	Follicular lymphoma	IV	yes	54	F
4	Follicular lymphoma	IIIA	yes	77	M
5	DLBCL	IV	no	48	M
6	DLBCL	IIIA	no	74	M
7	DLBCL	unknown	no	60	M
8	DLBCL	IVA	no	66	M
9	DLBCL	IVA	yes	43	F
10	DLBCL	IIIA	no	57	M
11	Follicular lymphoma	IVA	no	51	F
12	DLBCL	unknown	yes	70	M
13	Classic Hodgkin lymphma, small lymphocytic lymphoma	unknown	no	58	F
14	Burkitt lymphoma	IV	no	46	F
15	Mantle cell lymphoma	unknown	no	60	F
16	small lymphocytic lymphoma	unknown	no	74	F
17	DLBCL	IIIB	no	61	F
18	DLBCL	IVA	no	75	F
19	Follicular lymphoma	unknown	no	54	M
20	Follicular lymphoma	IVA	no	69	M
21	DLBCL	IVB	no	73	F
22	DLBCL	IV	no	39	F
32	DLBCL	IIIB	no	61	F
24	DLBCL	IIIA	no	59	F
25	DLBCL	IIIA	no	58	F
26	DLBCL	unknown	no	58	F
27	Follicular lymphoma	IIIB	no	47	F
28	DLBCL	IVA	no	55	F
29	DLBCL	IVA	no	66	M

Supplementary Table 2. The primers used for the quantitative qPCR**.**

Name	Sequence	
	Forward	Reverse
GAPDH	GGAGCGAGATCCCTCCAAAAT	GGCTGTTGTCATACTTCTCATGG
FOXM1	AGACCTGTGCAGATGGTGAG	CTGATGGTCTCG AAGGCTCC
C-MYC	CGTCTCCACACATCAGCACAA	TCTTGGCAGCAGGATAGTCCTT
cyclin A2	TTCACACATACCTTAGGGAAATGG	AGCCAAATGCAGGGTCTCAT
cyclin D1	CATCTACACCGACAACTCCATC	GTTCAATGAAATCGTGCGGG
cyclin E1	GTCCTGGCTGAATGTATACATGC	CCCTATTTTGTTCAGACAACAT
cyclin E2	AACTACCCAGGATGTCAAAAAAAGA	ATCAGGCAAAGGTGAAGGATTAATA
CDK1	TTCAGGATGTGCTTATGCAGGA	CTTCTAGAGTGACAAAACACAATCCC
CDK2	CCGCCTGGACACTGAGACT	GTGGAGGACCCGATGAGA
CDC25	CTACTCATCCCTGCCCTCTG	TCAAACCCGTAACACAGCAA
cyclin B1	TAAGATTGGAGAGGTTGATGTCGA	CCAGGTGCTGCATAACTGGA
cyclin B2	ATGTGACTATTAGGCGAACT	AGAGCAAGGCATCAGAAA

Supplementary Table 3. The IC50 values of RL, SC-1 and Karpas 422 cells treated with chiglitazar

Cell lines	Agents	24 h		48 h	
		IC50 (μM)	95% Confidence intervals (μM)	IC50 (μM)	95% Confidence intervals (μM)
RL	Chiglitazar	34.74	33.54 to 36.12	29.32	27.63 to 31.26
SC-1	Chiglitazar	37.97	35.17 to 41.53	25.35	23.25 to 27.69
Karpas 422	Chiglitazar	41.76	38.96 to 45.30	19.22	17.88 to 20.68
